# Temporal trends in time toxicity of R-CHOP: a nationwide hospital-based database analysis in Japan

**DOI:** 10.1007/s00520-025-09335-7

**Published:** 2025-03-18

**Authors:** Hiroaki Araie, Tomohisa Seki, Akira Okada, Toshimasa Yamauchi, Masaomi Nangaku, Takashi Kadowaki, Kazuhiko Ohe, Takahiro Yamauchi, Satoko Yamaguchi

**Affiliations:** 1 Department of Hematology and Oncology, Faculty of Medical Sciences, University of Fukui, Fukui, Japan; 2 Department of Prevention of Diabetes and Lifestyle-Related Diseases, Graduate School of Medicine, The University of Tokyo, 7-3-1, Hongo, Bunkyo-ku, Tokyo, 113-8655 Japan; 3 Department of Healthcare Information Management, The University of Tokyo Hospital, Tokyo, Japan; 4 Department of Diabetes and Metabolism, Graduate School of Medicine, The University of Tokyo, Tokyo, Japan; 5 Division of Nephrology and Endocrinology, Graduate School of Medicine, The University of Tokyo, Tokyo, Japan; 6 Toranomon Hospital, Tokyo, Japan; 7 Department of Bio-medical Informatics, Graduate School of Medicine, The University of Tokyo, Tokyo, Japan

**Keywords:** Time toxicity, R-CHOP regimen, B-cell lymphoma, Supportive care

## Abstract

**Purpose:**

While the prognosis of patients with cancer has improved, the time burden of treatment has recently been recognized as time toxicity; although, the actual clinical situation remains largely unexplored. This retrospective study aimed to elucidate the time toxicity of rituximab, cyclophosphamide, doxorubicin, vincristine, and prednisone (R-CHOP) in patients with B-cell lymphoma and the factors influencing it.

**Methods:**

We used a nationwide hospital-based database between January 2010 and November 2021 in Japan. We extracted the claims data of patients with diffuse large B-cell lymphoma and follicular lymphoma who were hospitalized and/or visited hospitals for chemotherapy.

**Results:**

Among the 7760 R-CHOP administered to 2006 patients, the rate of outpatient therapy increased over time (2010–2015: 17.9%; 2016–2021: 31.8%). In 2016, the median length of hospitalization was the shortest at 13 days (IQR 8–19), which coincided with the peak use of pegylated granulocyte colony-stimulating factor (Peg-G-CSF) during hospitalization in 2015–2016, likely driven by changes in the insurance system. In multivariate analysis, the factors associated with longer hospital stays were older age and poor activities of daily living, whereas the use of Peg-G-CSF, a reduced-dose regimen, and treatment at cancer-designated hospitals were associated with shorter stays.

**Conclusion:**

The time toxicity of R-CHOP has improved and may be influenced by the patient’s condition, adequate supportive care, changes in the insurance system, and center-specific treatment proficiency.

**Supplementary Information:**

The online version contains supplementary material available at 10.1007/s00520-025-09335-7.

## Introduction

Approximately two decades have elapsed since rituximab, cyclophosphamide, doxorubicin, vincristine, and prednisone (R-CHOP) were established as the standard treatment for B-cell lymphoma [1, 2]. During this period, several significant developments occurred in this field. The advent of pegylated granulocyte colony-stimulating factor (Peg-G-CSF) has allowed for more effective management of febrile neutropenia [3]. While several studies have demonstrated the impact of relative dose intensity (RDI) on treatment efficacy [4], reduced dose regimens have shown efficacy in older patients and have been widely adopted [5–7]. Although substantial evidence has been accumulated to improve the therapeutic efficacy of R-CHOP, the practical clinical choice of these supportive therapies and dose reductions in older patients varies widely across countries and regions with different patient backgrounds and healthcare systems [8–11]. Consequently, the real-world R-CHOP practices vary.

While survival and physical adverse events are typically the primary endpoints in clinical trials, recent years have seen growing recognition that the time patients spend in hospitals and outpatient visits is a significant treatment-related toxicity, referred to as “time toxicity” [12–15]. R-CHOP often requires inpatient administration, particularly during the first cycle of treatment, due to impaired performance status at diagnosis and/or the need to manage potential complications, including tumor lysis syndrome, febrile neutropenia, infusion reactions, or, less commonly, organ dysfunction or perforation due to mass effect [16, 17]. Prolonged hospitalization should be avoided in malignant lymphoma because more than half of the patients are over 65 years of age [18], which can adversely affect cognitive function and activities of daily living (ADLs) [19, 20]. Even for younger patients, interruptions in education or employment due to hospitalization or frequent outpatient visits can affect their quality of life and cause social distress [21]. While information on time toxicity is critical for patients to fully understand the overall treatment landscape and make informed decisions regarding R-CHOP, it has not yet been systematically evaluated.

Japan, a nation with one of the highest life expectancies in the world, has a comprehensive national health insurance system that allows the majority of patients, from the young to the older, to receive standard chemotherapy and supportive care without financial limitations [22, 23]. Thus, a substantial body of long-term real-world data on R-CHOP has been accumulated [23]. This study aimed to elucidate the actual time toxicity experienced by patients undergoing R-CHOP and to identify factors that exacerbate or mitigate this toxicity using a large hospital database.

## Patients and methods

### Data source

Our study used a hospital-based dataset purchased from Medical Data Vision (MDV, Tokyo, Japan). This comprehensive database aggregates claims information submitted by healthcare facilities across the country that use the Diagnosis and Procedure Combination (DPC) system for reimbursement purposes [22–24]. Currently, approximately 90% of all acute care hospitals in Japan use the DPC system, and the medical practices within these hospitals reflect Japan's approach to acute care medicine [25]. The database contains information such as age, sex, body height, body weight, diagnosis codes (according to the International Classification of Diseases, 10th Revision [ICD-10]) [25], medical procedures, drug prescriptions, and the status of cancer-designated hospitals. For hospitalized patients, data on admission and discharge dates, purpose of admission, outcomes, and Barthel index [26] were also available. We did not actively recruit patients for this study because we used an existing database. No identifiable or personal information was collected. Due to the anonymized nature of the data, informed consent was not required. This study was conducted according to the Declaration of Helsinki and approved by the Institutional Review Board of the Graduate School of Medicine of the University of Tokyo (2018030NI).

### Study design and population

Using the MDV database, we extracted the claims data of diffuse large B-cell lymphoma (DLBCL) and follicular lymphoma patients who were hospitalized and/or visited 49 anonymized hospitals (<200 beds, nine hospitals; 200–499 beds, 30 hospitals; and ≥500 beds, ten hospitals) for chemotherapy between January 1, 2010 and November 30, 2021. The inclusion criteria were as follows: (1) those diagnosed with diffuse large B-cell lymphoma (ICD-10 codes, C82) or follicular lymphoma (ICD-10 codes, C83.3) and (2) those who received R-CHOP-like regimens, such as R-CHOP, R-THP-COP (rituximab, pirarubicin, cyclophosphamide, vincristine, and prednisone) [27], or R-CVP (rituximab, cyclophosphamide, vincristine, and prednisone) [28].

### Study variables

We extracted data on the following variables: age, sex, body surface area (BSA) calculated using the Du Bois method, body mass index (BMI), Charlson comorbidity index (CCI), Barthel index, status of cancer-designated hospitals, use of Peg-G-CSF, and the RDI of R-CHOP. We divided BMI into three groups: under 18.5, 18.5 to 24.9, and 25.0 and higher, with a BMI of 22 kg/m^2^ set as the reference because it is considered an “ideal BMI” among the Japanese population [29]. The CCI, a weighted measure of comorbidities used to predict in-hospital mortality, was calculated based on admission diagnoses [30]. Since two points are always assigned due to the ubiquitous presence of malignant lymphoma in the patient cohort, the CCI was stratified into three categories according to a previous study: mild (CCI score of 2), moderate (CCI scores of 3–4), and severe (CCI scores ≥5) [31]. The RDI was calculated using height, weight, BSA, and amounts of vials of cyclophosphamide and daunorubicin, and we set the threshold at 80%, according to previous studies [4]. Among the various ADL evaluation tools, the Barthel index is widely used in oncology settings [26]. Japanese hospitals are required to assess and record ADLs in patients using the Barthel index on admission for inpatient care, and the corresponding scores are included in administrative claims data. We defined a full score (score: 100) as no disability and a score <100 as a disability. The cost analysis is presented based on an average exchange rate of ¥103 Japanese yen per US dollar, which represents the average exchange rate between the two currencies during the period from 2010 to 2021.

### Study outcomes

The analyses were conducted as follows: (1) implementation rates of R-CHOP and similar chemotherapy regimens, (2) trends in inpatient versus outpatient administration of R-CHOP, (3) duration of hospitalization for inpatient R-CHOP administration, (4) factors associated with hospital stay duration, (5) costs of hospitalization for inpatient R-CHOP administration, (6) broad-spectrum antimicrobial usage during R-CHOP hospitalization (fourth-generation cephalosporins, piperacillin–tazobactam, carbapenems; excluding prophylactic administration of new quinolone antimicrobials), and (7) frequency of outpatient visits for R-CHOP.

### Statistical analysis

Descriptive statistics were used to characterize the study population and outcomes. Temporal trends were analyzed by comparing the data from the early (2010–2015) and late (2016–2021) periods. Additionally, the Cochran–Armitage trend test was used for categorical variables, and the Mann–Kendall test was used for continuous variables to assess annual trends in outcomes. In the analysis of the length of hospital stay, patients who experienced in-hospital mortality were excluded because the rate of treatment-related mortality due to R-CHOP therapy was low [32] and the purpose of our analysis was to determine the actual length of hospital stay in patients who survived. The association between the length of hospital stay and cost was assessed using Pearson’s cumulative correlation coefficient. For the multivariate analysis, quantile regression models were used to evaluate the factors influencing the median length of hospital stay for cases in the later period. This approach was chosen to assess the impact of the covariates on the median values of these outcomes, thereby providing a more robust analysis in the presence of potential outliers or skewed distributions. The covariates included in the models were age (divided by the median), sex, BMI categories, CCI categories, ADLs categories, cancer-designated hospital status, Peg-G-CSF use, and R-CHOP RDI. The early period includes both the pre- and post-introduction phases of Peg-G-CSF, which introduces a significant source of bias in the multivariate analysis. Therefore, we limited the presentation of the multivariate analysis results to the later period. For sensitivity analysis, the first treatment recorded for a patient who had received six or more courses of R-CHOP or one of its similar regimens was defined as the first course, and analyses were performed for the first treatment and the second and subsequent courses. Two-sided *P*-values < 0.05 were considered statistically significant. All analyses were performed using the R software (version 4.1.1; R Foundation for Statistical Computing; Vienna, Austria). We used Claude 3.5 Sonnet to proofread the manuscript. All the revisions have been carefully reviewed and verified by the authors.

## Results

### Implementation rates of R-CHOP and similar chemotherapy regimens

Between January 2010 and November 2021, we identified 10,116 administrations of R-CHOP-like regimens (R-CHOP, R-THP-COP, and R-CVP) in 2599 patients with DLBCL and FL (Supplementary Fig. 1). R-CHOP was the most frequently used regimen (7760 administrations, 76.7%), followed by R-THP-COP (1674 administrations, 16.6%), and R-CVP (682 administrations, 6.7%) (Supplementary Fig. 2A). Sensitivity analysis limited to the first-cycle treatments showed a similar distribution of regimens (R-CHOP, 81.5% [657 cases]; R-THP-COP, 13.3% [107 cases]; and R-CVP, 5.2% [42 cases]; Supplementary Fig. 2B). Comparing the early period (2010–2015) with the late period (2016–2021), the median age of patients receiving R-CHOP changed from 65 years (interquartile range [IQR] 58–73) to 70 years (IQR 63–77), with the implementation rate increasing from 74.4 to 78.2%. For the R-THP-COP, the median patient age changed from 77 years (IQR 73–81) to 80 years (IQR 74–84), while the implementation rate decreased from 20.0 to 14.3%. For R-CVP, the median patient age changed from 79 years (IQR 67.5–85) to 77 years (IQR 69–84), with implementation rates of 5.6% and 7.5%, respectively.

### Inpatient vs. outpatient administration of R-CHOP

**Fig. 1:**
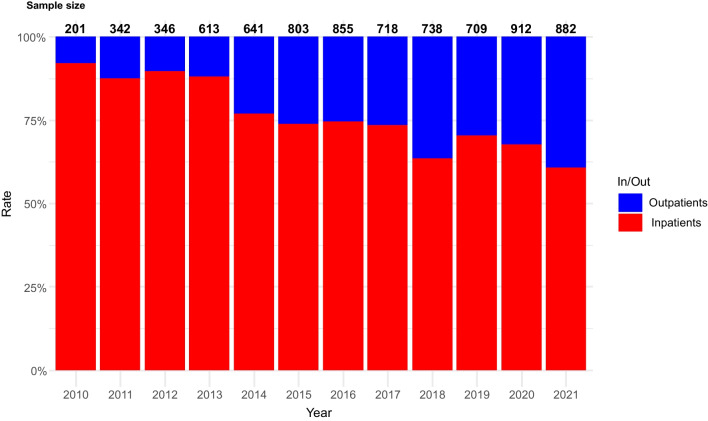
Temporal trends in the use of R-CHOP and R-CHOP-like chemotherapy regimens

Figure 1 shows the temporal trends in inpatient versus outpatient R-CHOP administration. Outpatient administration showed a gradual increase over time (early period, 17.9%; late period, 31.8%; *Z* = 16.5; *P* for trend < 0.001). Sensitivity analysis limited to first-cycle treatments showed that 98.8% of the patients received inpatient treatment throughout the study period (Supplementary Fig. 3A and Supplementary Table 1). For the second and subsequent cycles, the trend was similar to the overall pattern, with outpatient administration increasing over time (early period, 17.5%; later period, 40.3%; *Z* = 16.7; *P* for trend < 0.001; Supplementary Fig. 3B).

### Patient characteristics and hospital stay duration and costs for inpatient R-CHOP

Table 1 shows the characteristics of 3980 inpatients administered R-CHOP. The median age was 69 years (IQR 62–76), and 82.2% of patients were diagnosed with DLBCL. A total of 10.7% of patients exhibited disabilities in ADLs, 23.1% had moderate CCI, and 11.6% had severe CCI. A total of 77.2% of patients received treatment at cancer-designated hospitals. Dose reductions were implemented in 18.5% and 30.3% of the patients treated with cyclophosphamide and doxorubicin, respectively. Comparing early period with later period, patient age increased (median 64 years [IQR 58–72] vs. 70 years [IQR 63–78]), as did the proportion of patients receiving reduced doses (cyclophosphamide, 14.1% vs. 20.6%; doxorubicin, 23.1% vs. 33.6%). Post-2016, the age-specific reduction rates of R-CHOP with either cyclophosphamide, doxorubicin, or both were as follows: <65 years, 8.3%; 65–69 years, 11.7%; 70–74 years, 30.1%; 75–79 years, 57.1%; and 80–84 years, 89.2%.

**Table 1:** Patient status of R-CHOP therapy administered at each inpatient admission

	Overall	2010 to 2015	2016 to 2021
*n*	3980	1264	2716
Age (median [IQR])	69 (62, 76)	64 (58, 72)	70 [63, 78]
Female (%)	1921 (48.3)	637 (50.4)	1284 (47.3)
BMI (median [IQR])	22.1 (19.9, 24.50	22.3 (20.0, 24.6)	22.0 (19.9, 24.5)
BSA (median [IQR])	1.56 (1.43, 1.71)	1.56 (1.45, 1.71)	1.56 (1.42, 1.71)
Disease category			
DLBCL	3270 (82.2)	899 (81.1)	2371 (87.3)
FL	710 (17.8)	365 (28.9)	345 (12.7)
CCI (%)			
Mild	2578 (64.8)	875 (69.2)	1703 (62.7)
Moderate	919 (23.1)	248 (19.6)	671 (24.7)
Severe	462 (11.6)	125 (9.9)	337 (12.4)
Not available	21 (0.5)	16 (1.3)	5 (0.2)
ADL (%)			
No disability	3448 (86.6)	1120 (88.6)	2328 (85.7)
Disability	427 (10.7)	92 (7.3)	335 (12.3)
Not available	105 (2.6)	52 (4.1)	53 (2.0)
Cancer-designated hospital (%)	3072 (77.2)	1083 (85.7)	1989 (73.2)
Dose of cyclophosphamide (%)			
No dose reduction	3221 (80.9)	1069 (84.6)	2152 (79.2)
Dose reduction	738 (18.5)	178 (14.1)	560 (20.6)
Not available	21 (0.5)	17 (1.3)	4 (0.1)
Dose of doxorubicin (%)			
No dose reduction	2754 (69.2)	955 (75.6)	1799 (66.2)
Dose reduction	1205 (30.3)	292 (23.1)	913 (33.6)
Not available	21 (0.5)	17 (1.3)	4 (0.1)

*ADL* activities of daily living, *BMI* body mass index, *BSA* body surface area, *CCI* Charlson comorbidity index, *DLBCL* diffuse large B-cell lymphoma, *FL* follicular lymphoma

The CCI was stratified into three categories: mild (CCI score of 2), moderate (CCI scores of 3–4), and severe (CCI scores ≥5)

**Fig. 2:**
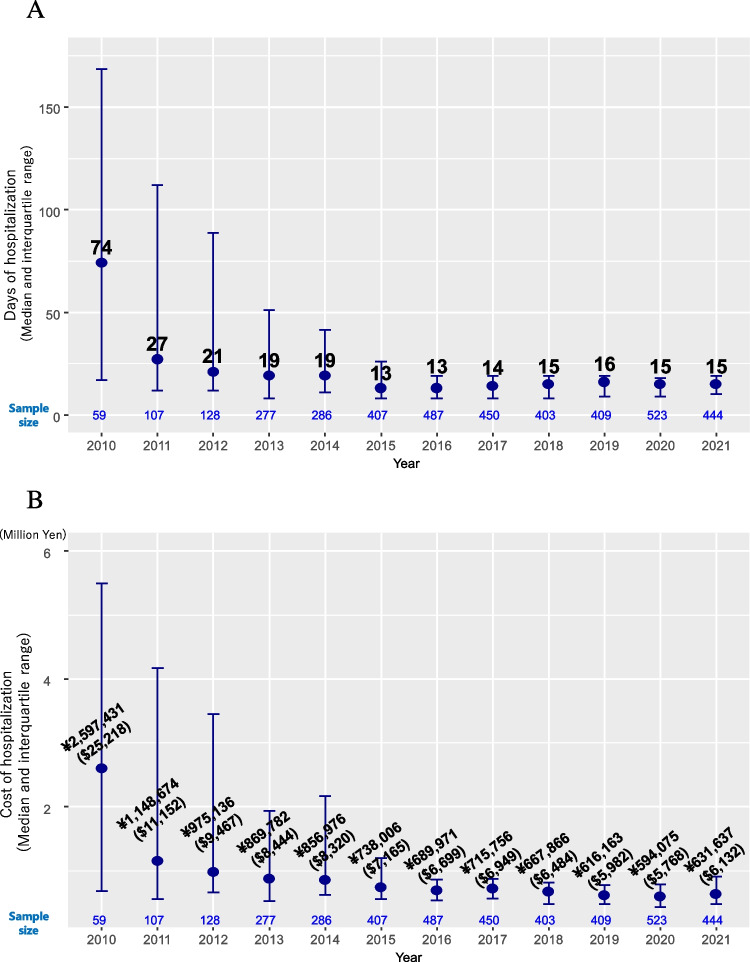
Temporal trends in the length of hospital stay and costs for inpatient R-CHOP. **A** Median length of hospital stay. **B** The median number of hospitalization costs

The median length of hospital stay decreased from 74 days (IQR 17–168.5) in 2010 to 13 days (IQR 8–19) in 2016, reflecting a shift from multiple R-CHOP cycles per admission to a single cycle (Fig. 2A). From 2017, despite maintaining single-cycle admissions, the median length of stay increased slightly to 15 days (IQR 10–19) by 2021. After the introduction of Peg-G-CSF in Japan in November 2014, its inpatient use peaked at 34.9% in 2015 and 23.7% in 2016 and then decreased to less than 10% from 2018 (Supplementary Table 2). Post-2016, the median length of stay showed a decreasing trend from the first cycle (17 days, IQR 13–28) to the sixth cycle (11 days, IQR 7–17). However, the median length of stay was longer for the seventh (14 days, IQR 8–18) and eighth (14.5 days, IQR 8–19) cycles compared to the sixth cycles (Supplementary Table 3). To evaluate the potential impact of tumor lysis syndrome on the length of hospital stay, we analyzed the 53 cases who received rasburicase, a therapeutic agent administered to patients at high risk for or with severe tumor lysis syndrome, and found that the median length of stay was 29 days (IQR 20–54).

Multivariable analysis of the median hospital length of stay for cases in the late period showed that the use of Peg-G-CSF, R-CHOP dose reduction, and treatment in cancer-designated hospitals were independently associated with a reduction in the length of stay of at least 1 day. Conversely, older age (≥70 years) and disability of ADLs were independently associated with an increase in the length of stay by at least 1 day (Table 2 and Supplementary Fig. 4). No multicollinearity was detected among the variables included in the multivariate analysis.

**Table 2:** Multivariate analysis of median length of hospital stay

	Days	Standard error	*P*
(Intercept)	17.0	0.43	
Age ≥70 years	1.0	0.22	<0.001
Female	0.0	0.22	>0.99
BMI 18.5–24.9 (kg/m^2^)	0.0	0.39	>0.99
BMI ≥25 kg/m^2^	0.0	0.41	>0.99
CCI moderate	0.0	0.33	>0.99
CCI severe	1.0	0.52	0.054
Disability of ADL	4.0	0.60	<0.001
Cancer-designated hospital	−4.0	0.24	<0.001
Use of Peg-G-CSF	−5.0	0.26	<0.001
Dose reduction	−2.0	0.30	<0.001

*Peg-G-CSF* pegylated granulocyte colony-stimulating factor

Other abbreviations are listed in Tables 1

The median hospitalization cost decreased annually, reaching ¥631,637 Japanese yen ($6132) (IQR ¥475,721 ($4619) – ¥910,768 ($8842)) by 2021 (*Z* = −4.0, *P* for trend < 0.001; Fig. 2B). A correlation was observed between the length of hospital stay and the associated hospitalization costs (*r* = 0.94; Supplementary Fig. 5).

The use of broad-spectrum antibiotics during hospitalization differed by patient characteristics (Supplementary Table 4): 1.6% (95% CI 0.8–3.1) for patients < 70 years without dose reduction, Peg-G-CSF, or non-Peg-G-CSF (*n* = 540); 7.0% (95% CI 2.3–15.6) for patients ≥70 years without dose reduction but with Peg-G-CSF (*n* = 71); and 7.8% (95% CI 3.2–15.4) for patients ≥70 years with both dose reduction and Peg-G-CSF (*n* = 90).

### Outpatient visit frequency for R-CHOP

**Fig. 3:**
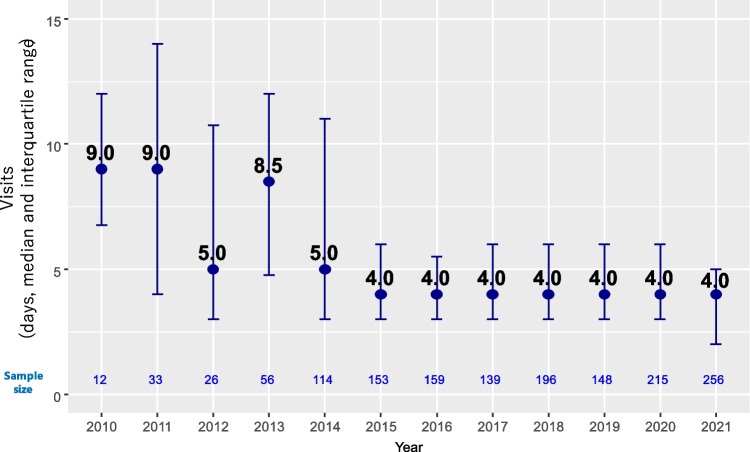
Median number of visits per month for R-CHOP in the outpatient setting

Figure 3 shows the number of visits per month for a total of 1507 months, excluding the months of outpatient treatment and hospitalization in the same month, for 616 patients who received R-CHOP in the outpatient setting. The median number of monthly outpatient visits decreased from 6 days (IQR 4–12) during the pre-Peg-G-CSF era of 2010–2014 to 4 days (IQR 3–6) in the post-Peg-G-CSF implementation period of 2015–2021.

## Discussion

The use of R-CHOP showed a progressive increase over time, extending to older patients, suggesting widespread adoption of dose-reduction regimens. Temporal analysis revealed a decrease in the length of hospital stay, decreased frequency of outpatient visits, and an increased proportion of R-CHOP administrations in outpatient settings. Variables associated with longer hospital stays were older age and disability in ADLs, while the use of Peg-G-CSF, R-CHOP dose reduction, and treatment at cancer-designated hospitals were associated with shorter stays.

Japan’s comprehensive national health insurance system includes a cost-sharing mechanism based on age and income, which facilitates broad access to standard therapeutic interventions without significant financial barriers [22]. Therefore, the threshold for extended hospitalization during chemotherapy remains relatively low because of modest out-of-pocket expenses for patients [33, 34]. Conversely, healthcare providers bill insurers for inpatient costs using the DPC system [24]. Given the typically low per-diem reimbursement rates, extended hospital stays are often necessary to offset expenses associated with high-cost drugs. The introduction of Peg-G-CSF in Japan in November 2014 [35] initially circumvented the constraints of the DPC system, allowing easy inpatient administration under a fee-for-service reimbursement system. However, its subsequent inclusion in the DPC system in April 2016 made the inpatient use of Peg-G-CSF financially unsustainable, resulting in a precipitous decline to less than 10% after 2018, as confirmed in this research. In other words, it was inferred that these changes in the insurance system likely contributed to the decreased use of Peg-G-CSF and the subsequent increase in hospitalization length, despite the shortest hospitalization length occurring in 2015 and 2016, when Peg-G-CSF use was higher. The COVID-19 pandemic has profoundly changed the healthcare landscape since 2020, potentially affecting the length of hospital stays. However, our analysis of the same dataset showed that rates of chemotherapy hospitalizations and outpatient chemotherapy treatments for cancer patients remained relatively stable compared to pre-pandemic levels [36].

Various derivative regimens of R-CHOP have been developed based on the severity of the underlying malignancy and patient comorbidities [37–40]. In Japan, R-THP-COP has been widely used as an alternative regimen, especially in geriatric patients, due to its reported non-inferiority to R-CHOP [27, 41]. However, its use has been declining in recent years, possibly due to the global establishment of evidence supporting dose-reducing regimens in older patients, such as R-miniCHOP [5]. Additionally, while maintaining the dose intensity of R-CHOP is critical for therapeutic efficacy in patients younger than 80 years with a good performance status [4], this study showed that in real-world data, 30.1% of patients aged 70–74 years and 57.1% of patients aged 75–79 years underwent dose reduction. Although whether patients who receive dose reductions truly require such modifications remains unclear, comprehensive geriatric assessment tools are being considered for pretreatment evaluation to objectively identify candidates for standard therapy [42, 43]. Widespread implementation of these tools is anticipated to lead to more appropriate patient stratification for dose-reduction regimens.

This study found that the length of hospital stay was shorter in cancer-designated hospitals. Previous research has shown that treatment outcomes in various malignancies differ depending on the proficiency of treatment at different institutions [44, 45]. On the other hand, to the best of our knowledge, this is the first report to show that differences between institutions also affect time toxicity. Differences in the length of hospital stay may be due to differences in the proficiency levels of the institutions in areas such as the efficiency of medical care through the introduction of clinical pathways, appropriate reduction of anticancer drugs for each patient, and implementation of supportive care. Additionally, cancer-designated hospitals typically function as high-volume centers requiring efficient patient admission, discharge, and bed management, which may have contributed to the observed reduction in hospital length of stay.

Complications of febrile neutropenia are factors prolonging hospitalization with R-CHOP [16]. In this study, the rate of the use of broad-spectrum antibiotics was 7% in patients older than 70 years when prophylaxis with Peg-G-CSF was used. This result was similar to those of recent clinical trials on R-CHOP [46]. Furthermore, the complication rate of FN in patients younger than 70 years without Peg-G-CSF or non-Peg-G-CSF was remarkably low (1.7%), suggesting that prolonged hospitalization to monitor the duration of the nadir may be unnecessary in many patients. This study did not evaluate cases in which non-Peg-G-CSF was administered alone because the dataset did not allow for differentiation between prophylactic and therapeutic use.

This study has several limitations. This retrospective analysis was limited by the lack of laboratory parameters, lymphoma staging data, and residential classification (urban or rural) in the database, which precluded the complete elimination of confounding variables. Furthermore, the limitation of the dataset to 49 centers introduces the possibility of selection bias. Additionally, the hospital-centered data collection methodology made it difficult to track patients who were transferred and subsequently treated at alternative medical facilities. Additionally, the data collection period, which ended in 2021, did not include the polatuzumab vedotin-R-CHP regimen, which is one of the standard treatments for DLBCL. The introduction of polatuzumab vedotin in Japan in May 2021 is unlikely to have had a significant impact on R-CHOP selection, except in the second half of 2021.

In conclusion, the length of hospital stay for R-CHOP in Japan has decreased, and the proportion of R-CHOP administered on an outpatient basis has increased, resulting in reduced time toxicity. In addition to the influence of ADLs as a patient factor, factors such as the use of Peg-G-CSF and a reduction in the chemotherapy dose may also influence the length of hospital stay. Differences in treatment strategies among institutions may also have a significant impact. Notably, real-world evidence shows that changes in the insurance system have significantly hindered the use of Peg-G-CSF, with adverse consequences of time toxicity. This study highlights that when considering strategies to improve time toxicity, it is imperative to not only calibrate patient-specific factors and treatment modalities but also to consider the impact of healthcare policy.

## Supplementary Information

Below is the link to the electronic supplementary material.

Supplementary file1 (DOCX 4.11 MB)

## Data Availability

No datasets were generated or analysed during the current study.
