# Supplementary material for: Temporal trends in time toxicity of R-CHOP: a nationwide hospital-based database analysis in Japan
Source: Support Care Cancer. 2025 Mar 18;33(4):293. doi: 10.1007/s00520-025-09335-7 (PMC11913952; doi:10.1007/s00520-025-09335-7)
Supplement: Supplementary file 1 — Supplementary file1 (DOCX 4.11 MB) [file 520_2025_9335_MOESM1_ESM.docx]

**Title:** Temporal trends in time toxicity of R-CHOP: a nationwide hospital-based database analysis in Japan

**Running head:** Temporal trends in time toxicity of R-CHOP in Japan

**Authors:** Hiroaki Araie^1,2,*^, Tomohisa Seki^3^, Akira Okada^2^, Toshimasa Yamauchi^4^, Masaomi Nangaku^5^, Takashi Kadowaki^2,4,6^, Kazuhiko Ohe^3,7^, Takahiro Yamauchi^1^, Satoko Yamaguchi^2,*^

**Affiliation**s

1. Department of Hematology and Oncology, Faculty of Medical Sciences, University of Fukui, Fukui, Japan.

2. Department of Prevention of Diabetes and Lifestyle-Related Diseases, Graduate School of Medicine, The University of Tokyo, Tokyo, Japan.

3. Department of Healthcare Information Management, The University of Tokyo Hospital, Tokyo, Japan.

4. Department of Diabetes and Metabolism, Graduate School of Medicine, The University of Tokyo, Tokyo, Japan

5. Division of Nephrology and Endocrinology, Graduate School of Medicine, The University of Tokyo, Tokyo, Japan

6. Toranomon Hospital, Tokyo, Japan

7. Department of Bio-medical Informatics, Graduate School of Medicine, The University of Tokyo, Tokyo, Japan.

**Corresponding author:**

Satoko Yamaguchi, address: Department of Prevention of Diabetes and Lifestyle-Related Diseases, Graduate School of Medicine, The University of Tokyo, 7-3-1, Hongo, Bunkyo-ku, Tokyo 113-8655, Japan E-mail: [syamaguc@m.u-tokyo.ac.jp](mailto:syamaguc@m.u-tokyo.ac.jp), phone number :+81-3-3815-5411

**Supplementary Figure Legends**

**Supplementary Figure 1.** Flowchart of the patient selection process

**Supplementary Figure 2.** (A) Temporal trends in inpatient versus outpatient administration of R-CHOP. (B) Sensitivity analysis was limited to first-cycle treatments with temporal trends in the use of R-CHOP and R-CHOP-like chemotherapy regimens

**Supplementary Figure 3.** Sensitivity analysis of temporal trends in inpatient versus outpatient R-CHOP administration. (A) First cycle treatment. (B) Second and subsequent cycle treatments

**Supplementary Figure 4.** Quantile regression coefficients with 95% confidence intervals for factors influencing (A) the length of hospital stay and (B) hospitalization costs. Each panel represents a different predictor variable and shows how its effect varies across different quantiles of the outcome distribution. The quantile (tau) represents different percentiles of the outcome distribution from 0 (minimum) to 1 (maximum)

**Supplementary Figure 5.** Scatterplot of the correlation between hospitalization days and costs


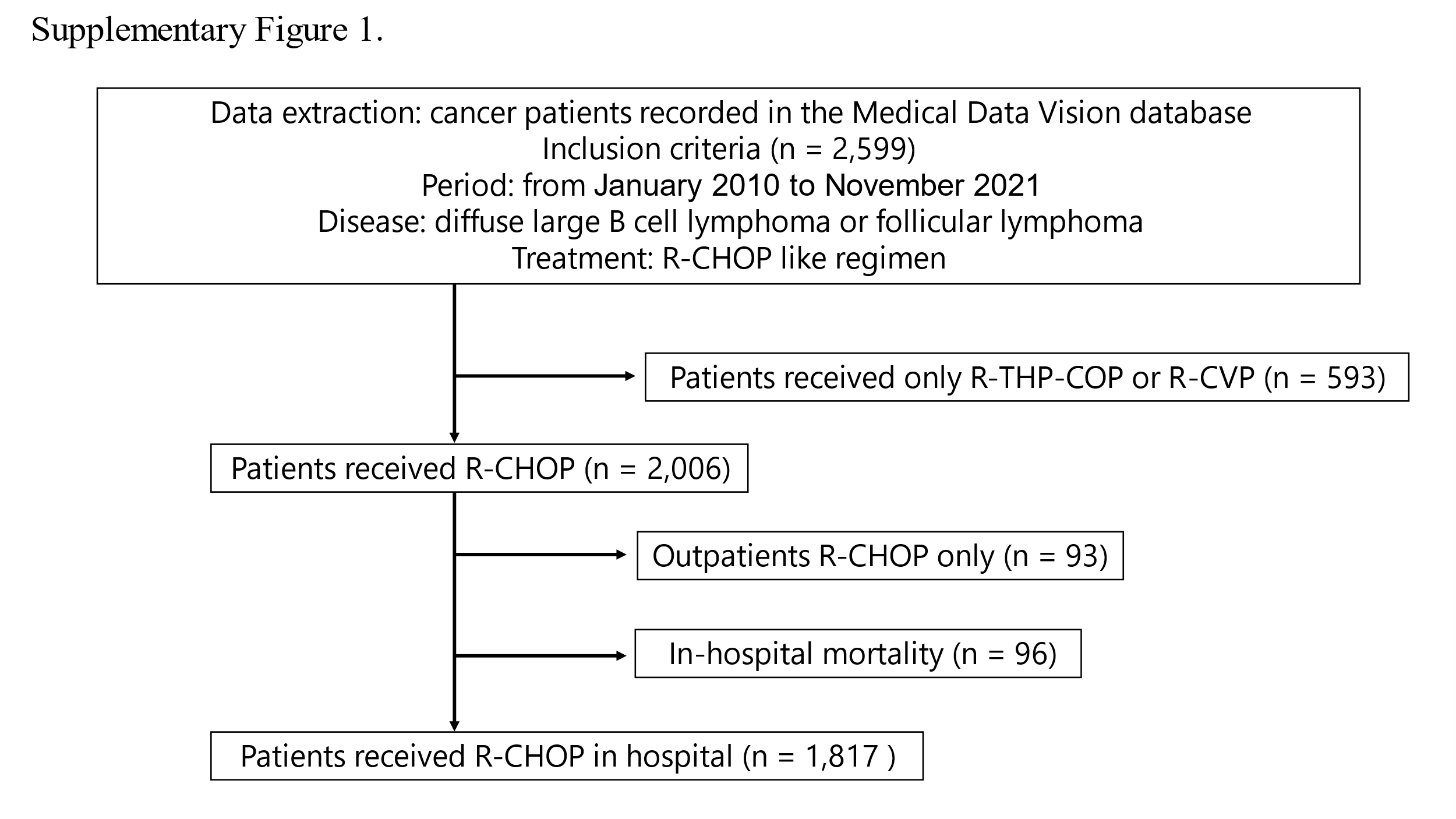


**Supplementary Figure 2**

**A**

**
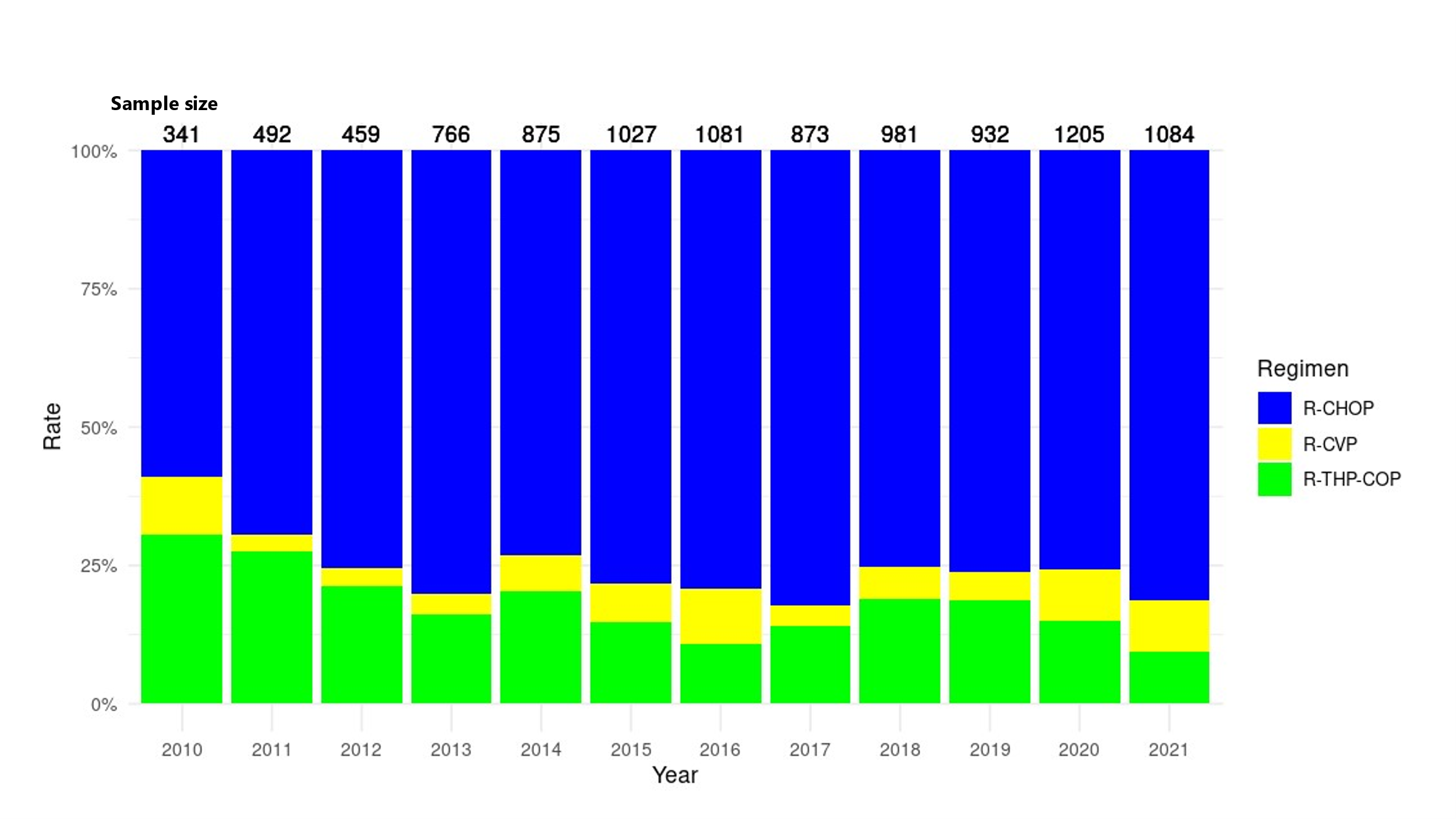
**

**B**

**
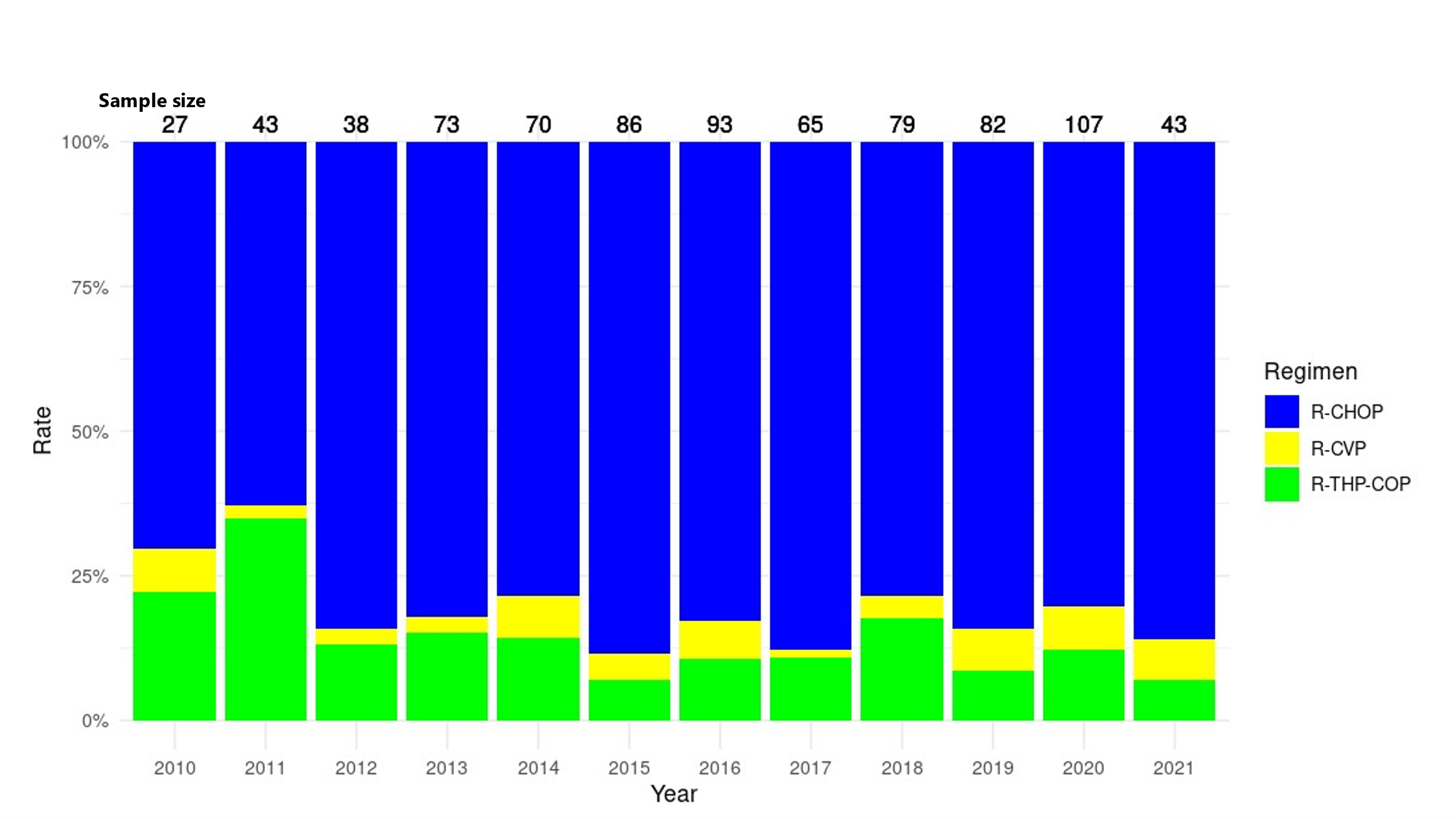
**


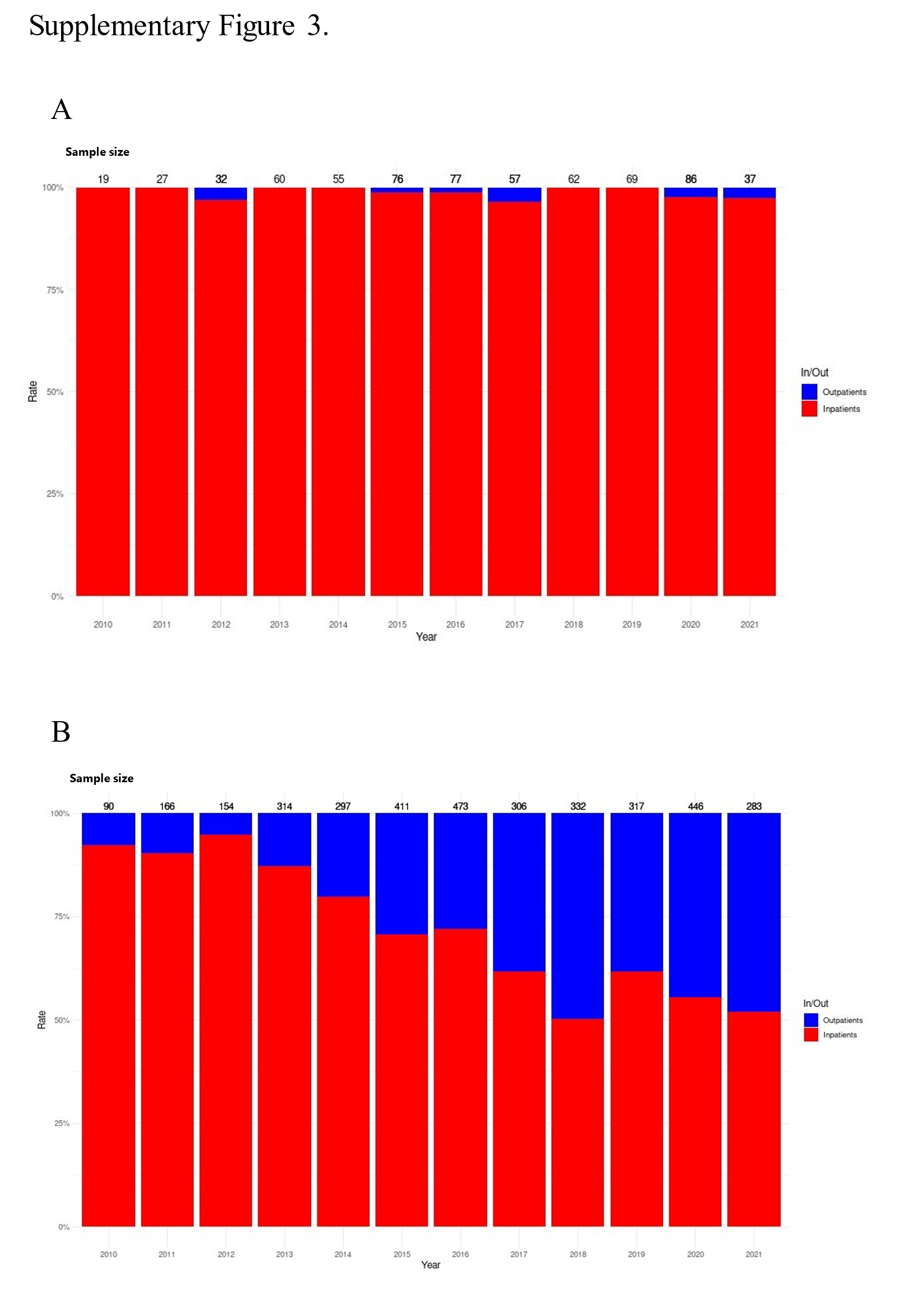


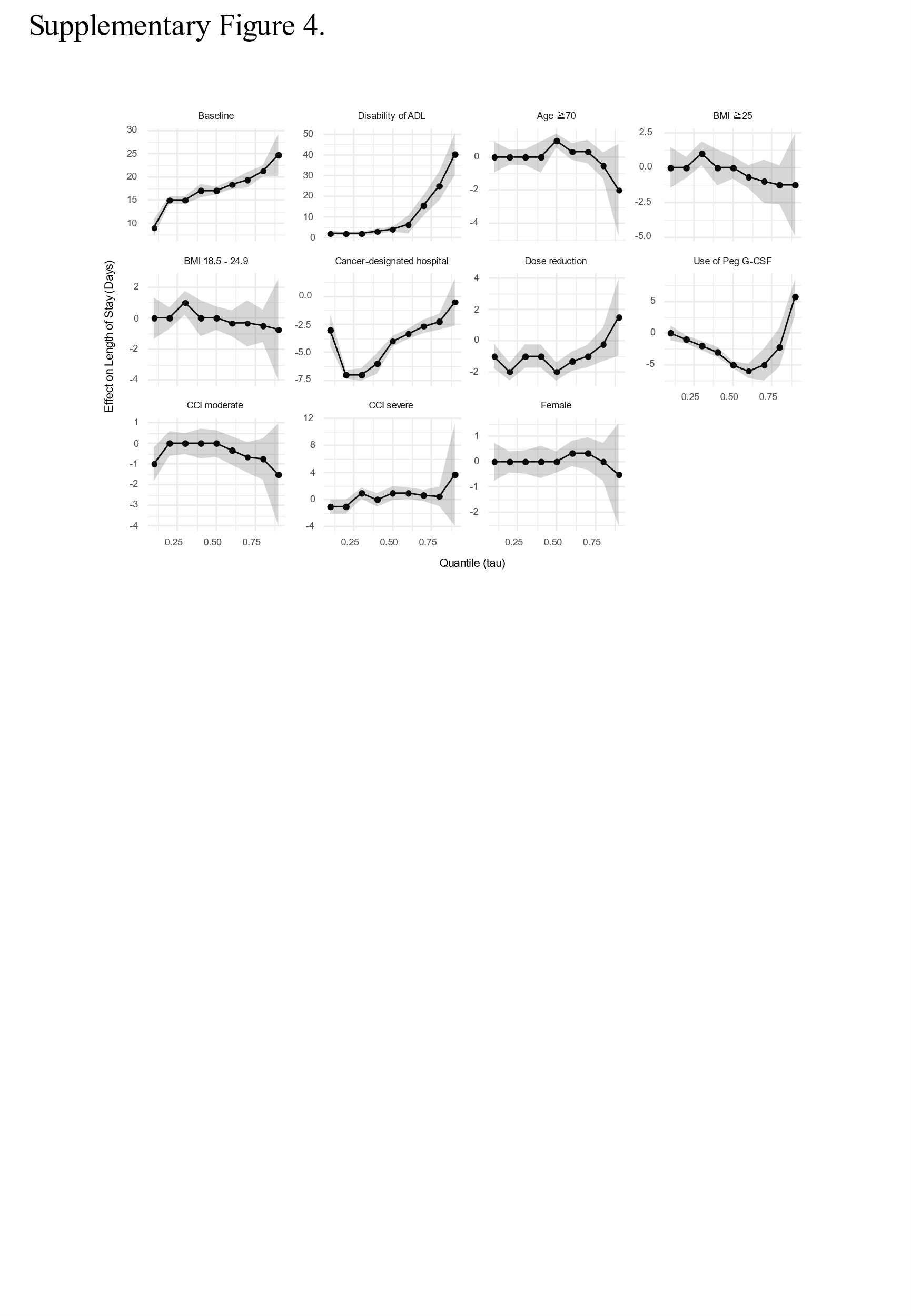


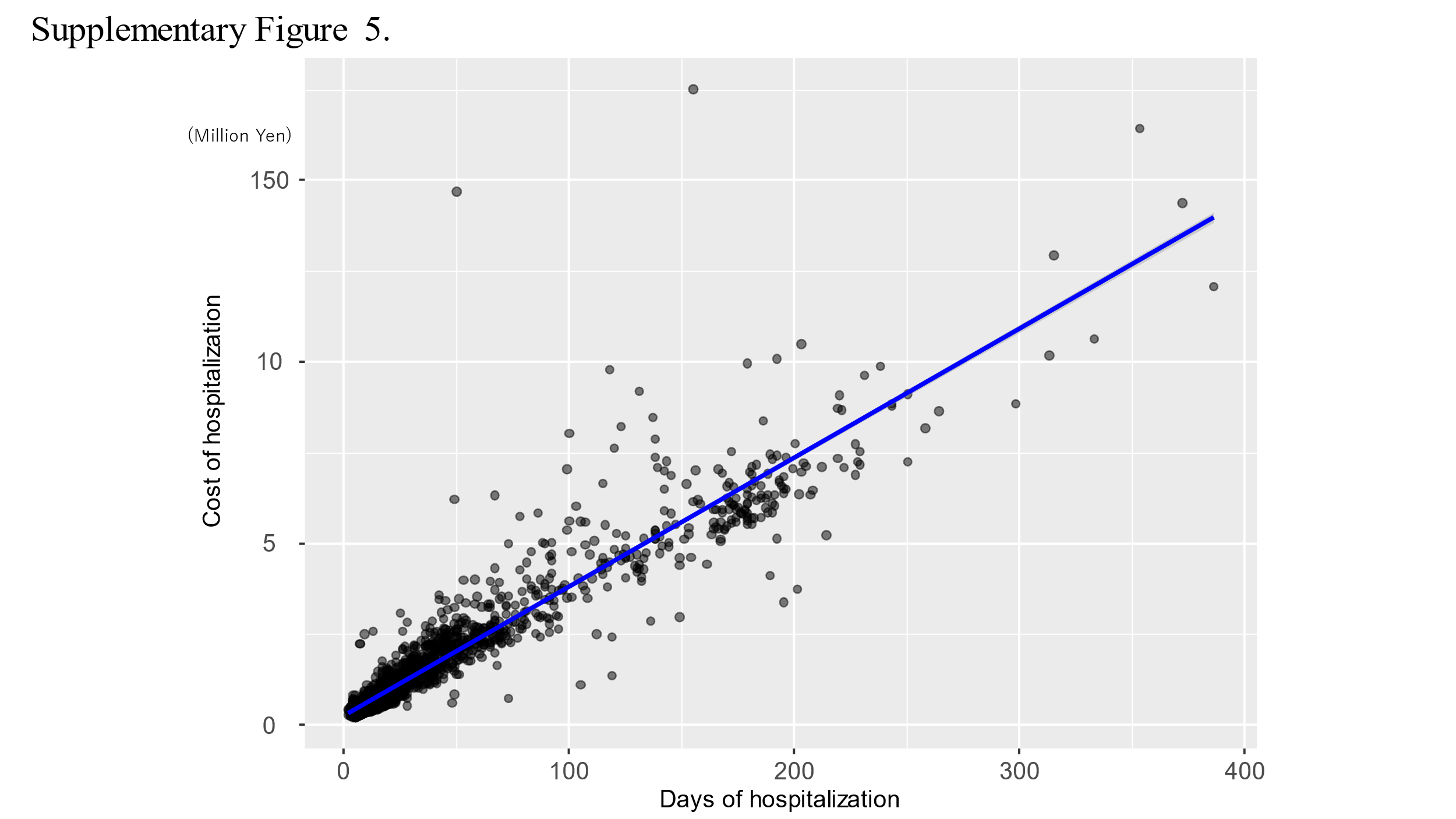


**Supplementary Table 1.** Patient status of R-CHOP therapy administered at first-cycle

|  |  |  |  |  |  |  |  |  |  |  |  |  |  |
| --- | --- | --- | --- | --- | --- | --- | --- | --- | --- | --- | --- | --- | --- |
|  | Overall | 2010 | 2011 | 2012 | 2013 | 2014 | 2015 | 2016 | 2017 | 2018 | 2019 | 2020 | 2021 |
| n | 588 | 19 | 25 | 32 | 58 | 49 | 68 | 66 | 43 | 56 | 61 | 76 | 35 |
| Age (median[IQR]) | 69[61,76] | 68[54,76] | 68[60,71] | 63[54,75] | 66[59,72] | 65[59,72] | 67[60,73] | 67[58,72] | 71[67,76] | 67[60,76] | 73[66,78] | 72[64,80] | 72[68,79] |
| Disease category |  |  |  |  |  |  |  |  |  |  |  |  |  |
| DLBCL | 487(82.8) | 15(78.9) | 14(56.0) | 25(78.1) | 46(79.3) | 35(71.4) | 49(72.1) | 56(84.8) | 37(86.0) | 52(92.9) | 57(93.4) | 66(92.1) | 68(88.6) |
| FL | 101(17.2) | 4(21.1) | 11(44.0) | 7(21.9) | 12(20.7) | 14(28.6) | 19(27.9) | 10(15.2) | 6(14.0) | 4(7.1) | 4(6.6) | 6(7.9) | 4(11.4) |
| Cancer-designated hospital (%) | 472(80.3) | 19(100.0) | 24(96.0) | 30(93.8) | 40(69.0) | 42(85.7) | 58(85.3) | 59(89.4) | 32(74.4) | 42(75.0) | 46(75.4) | 52(68.4) | 28(80.0) |
| Dose of cyclophosphamide (%) |  |  |  |  |  |  |  |  |  |  |  |  |  |
| No dose reduction | 461(78.4) | 13(68.4) | 19(76.0) | 22(68.8) | 51(87.9) | 40(81.6) | 52(76.5) | 56(84.8) | 33(76.7) | 49(87.5) | 47(77.0) | 54(71.1) | 25(71.4) |
| Dose reduction | 124(21.1) | 5(26.3) | 5(20.0) | 10(31.2) | 7(12.1) | 9(18.4) | 16(23.5) | 10(15.2) | 10(23.3) | 7(12.5) | 14(23.0) | 22(28.9) | 9(25.7) |
| Not available | 3(0.5) | 1(5.3) | 1(4.0) | 0(0.0) | 0(0.0) | 0(0.0) | 0(0.0) | 0(0.0) | 0(0.0) | 0(0.0) | 0(0.0) | 0(0.0) | 1(2.9) |
| Dose of doxorubicin (%) |  |  |  |  |  |  |  |  |  |  |  |  |  |
| No dose reduction | 396(67.3) | 12(63.2) | 16(64.0) | 20(62.5) | 46(79.3) | 39(79.6) | 47(69.1) | 48(72.7) | 30(69.8) | 38(67.9) | 34(55.7) | 46(60.5) | 20(57.1) |
| Dose reduction | 189(32.1) | 6(31.6) | 8(32.0) | 12(37.5) | 12(20.7) | 10(20.4) | 21(30.9) | 18(27.3) | 13(30.2) | 18(32.1) | 27(44.3) | 30(39.5) | 14(40.0) |
| Not available | 3(0.5) | 1(5.3) | 1(4.0) | 0(0.0) | 0(0.0) | 0(0.0) | 0(0.0) | 0(0.0) | 0(0.0) | 0(0.0) | 0(0.0) | 0(0.0) | 1(2.9) |

Abbreviations: DLBCL, Diffuse large B-cell lymphoma; FL, Follicular lymphoma.

**Supplementary Table 2.** Annual changes in Peg-G-CSF use during hospitalization

|  |  |  |  |
| --- | --- | --- | --- |
| Year | Total number of admissions | Use of Peg-G-CSF during hospitalization | Usage ratio |
| 2014 | 291 | 3 | 1.0% |
| 2015 | 416 | 145 | 34.9% |
| 2016 | 493 | 117 | 23.7% |
| 2017 | 461 | 72 | 15.6% |
| 2018 | 418 | 27 | 6.5% |
| 2019 | 432 | 11 | 2.5% |
| 2020 | 540 | 31 | 5.7% |
| 2021 | 461 | 35 | 7.6% |

Abbreviation: Peg-G-CSF, pegylated granulocyte colony-stimulating factor.

**Supplementary Table 3.** Length of hospital days for R-CHOP for each cycle

|  |  |  |
| --- | --- | --- |
| cycle | n | Length of hospital stay (median [IQR]) |
| 1 | 337 | 17 [13, 28] |
| 2 | 250 | 16 [9, 29.75] |
| 3 | 218 | 12 [7, 17.75] |
| 4 | 214 | 12 [7, 18] |
| 5 | 210 | 11.5 [7, 17] |
| 6 | 212 | 11 [7, 17] |
| 7 | 62 | 14 [8, 18] |
| 8 | 40 | 14.5 [8, 19] |

**Supplementary Table 4.** The use of broad-spectrum antibiotics during hospitalization differed according to the patient characteristics

|  |  |  |  |
| --- | --- | --- | --- |
| Patients | Total number of admissions | Use of broad-spectrum antibiotics | Ratio (95%CI) |
| <70 years without dose reduction, Peg-G-CSF, or non-Peg-G-CSF | 540 | 9 | 1.6% (0.8–3.1) |
| ≥70 years without dose reduction but with Peg-G-CSF | 71 | 5 | 7.0% (2.3–15.6) |
| ≥70 years with both dose reduction and Peg-G-CSF | 90 | 7 | 7.8% (3.2–15.4) |
